# PSYCHOSOCIAL PROTECTIVE FACTORS FOR COGNITIVE AGING: THE MORE THE BETTER

**DOI:** 10.1093/geroni/igae098.0508

**Published:** 2024-12-31

**Authors:** Morgan Taylor, Kylie Schiloski, Margie Lachman

**Affiliations:** Brandeis University, Waltham, Massachusetts, United States; Brandeis University, Waltham, Massachusetts, United States; Brandeis University, Waltham, Massachusetts, United States

## Abstract

Research demonstrates that demographic (e.g., age, education) and behavioral variables (e.g., physical activity, smoking) are related to individual differences in cognitive aging. Although some work has examined modifiable psychosocial factors (e.g., control beliefs, purpose in life, social contact) in relation to cognitive aging, they are typically examined separately. To examine their additive rather than net effects, we standardized and averaged three psychosocial factors to create a continuous composite of sense of control, purpose in life, and social support in a sample of adults (N = 2,497, ages 28-85) from the second (2004-2006) and third (2013-2014) waves of the Midlife in the United States study. Using multiple regression, we tested the composite as a predictor of 9 to 10 year changes in episodic memory and executive functioning. Results showed that those higher on the psychosocial composite demonstrated less decline in both cognitive measures, even after controlling for established demographic, health, and behavioral risk factors of cognitive aging. We then assigned participants a score of 0 if they were below the median or a 1 if equal to or above the median on each psychosocial factor, such that total scores varied from 0 to 3. Those high on all three factors were better able to maintain their cognitive health compared to those who had fewer. These findings suggest that interventions that focus on a combination of psychosocial factors could be protective in reducing the rate of cognitive decline in middle and older adulthood.

